# Growth of *Chitinophaga pinensis* on Plant Cell Wall Glycans and Characterisation of a Glycoside Hydrolase Family 27 β-l-Arabinopyranosidase Implicated in Arabinogalactan Utilisation

**DOI:** 10.1371/journal.pone.0139932

**Published:** 2015-10-08

**Authors:** Lauren S. McKee, Harry Brumer

**Affiliations:** 1 Division of Glycoscience, School of Biotechnology, Royal Institute of Technology (KTH), AlbaNova University Centre, 106 91, Stockholm, Sweden; 2 Wallenberg Wood Science Centre, Teknikringen 56–56, 100 44, Stockholm, Sweden; 3 Michael Smith Laboratories and Department of Chemistry, University of British Columbia, 2185 East Mall, Vancouver, V6T 1Z4, BC, Canada; INRA, FRANCE

## Abstract

The genome of the soil bacterium *Chitinophaga pinensis* encodes a diverse array of carbohydrate active enzymes, including nearly 200 representatives from over 50 glycoside hydrolase (GH) families, the enzymology of which is essentially unexplored. In light of this genetic potential, we reveal that *C*. *pinensis* has a broader saprophytic capacity to thrive on plant cell wall polysaccharides than previously reported, and specifically that secretion of β-l-arabinopyranosidase activity is induced during growth on arabinogalactan. We subsequently correlated this activity with the product of the Cpin_5740 gene, which encodes the sole member of glycoside hydrolase family 27 (GH27) in *C*. *pinensis*, *Cp*Arap27. Historically, GH27 is most commonly associated with α-d-galactopyranosidase and α-d-*N*-acetylgalactosaminidase activity. A new phylogenetic analysis of GH27 highlighted the likely importance of several conserved secondary structural features in determining substrate specificity and provides a predictive framework for identifying enzymes with the less common β-l-arabinopyranosidase activity.

## Introduction

Microorganisms with the capacity to selectively and efficiently degrade plant-derived carbohydrates are of great interest to research and industry as a source of new tools for the characterisation and degradation of plant biomass [1–7]. *Chitinophaga pinensis* is a motile, gram-negative bacterium that was originally characterised by its ability to utilise the eponymous insect and fungal polysaccharide chitin [8, 9]. However, *C*. *pinensis* was in fact isolated from pine forest leaf litter, an environment which would be expected to provide a rich source of plant cell wall glycans. More recently, the complete genome sequence of *C*. *pinensis*, generated as part of the Joint Genome Institute’s Genomic Encyclopedia of Bacteria and Archaea (GEBA) project [10], revealed a large variety of catabolic carbohydrate-active enzymes (CAZymes), including 193 glycoside hydrolases from 56 of the 130 known glycoside hydrolase (GH) families (see http://www.cazy.org/b1017.html) [11, 12]. The predicted diversity of these enzymes expands well beyond the handful of GH families implicated in chitin degradation. Indeed, only 10 of the GH enzymes encoded by this genome are predicted to act on chitin (chitinases and *N*-acetylglucosaminidases) [12]. Thus, it seems likely that *C*. *pinensis* has a wider ability than previously appreciated to grow on complex plant biomass polysaccharides in its native environment [8]. At the same time, the limited ability of this bacterium to grow on cellulose and starch suggests that *C*. *pinensis* may preferentially degrade the amorphous matrix glycans that are ubiquitous and abundant in plant cell walls. As such, the genome of *C*. *pinensis* constitutes a rich resource for the discovery of new CAZymes. Indeed, the CAZyme complement of *C*. *pinensis* ranks highly among other well-endowed Bacteroidetes from gut microbiota, which likewise must address a diversity of complex plant glycans [13].

In the present study, we explored this catalytic potential by surveying the growth of *C*. *pinensis* on a panel of purified polysaccharide substrates to reveal that this bacterium is in fact a prolific degrader of plant -derived glycans. Due to our continuing interest in the evolution of glycosidase diversity [5, 14–17], and in particular specificity and mechanism in glycoside hydrolase family 27 (GH27) [18, 19], we fully characterised the product of the sole GH27 gene in *C*. *pinensis*, Cpin_5740, secretion of which is induced during growth on arabinogalactan. Biochemical analysis of the recombinant wild-type enzyme, henceforth referred to as *Cp*Arap27 [20], and site-directed mutants showed that this enzyme is exquisitely specific for β-l-arabinopyranosides, *vis-a-vis* the well-known specificity of GH27 members for *galacto*-configured substrates. Building on previous work [21, 22], we incorporate these new data into an updated phylogenetic analysis of GH27 that includes the more recent discovery of β-l-arabinopyranosidase activity in the family, and assess the reliability of conserved secondary structural elements as predictors of enzyme activity.

## Experimental Procedures

### Carbohydrates

The 4-nitrophenyl glycoside substrates used in this study (Gal-α-PNP and l-Ara*p*-β-PNP) were purchased from Sigma-Aldrich. The polysaccharides beech wood xylan and Gum Arabic (acacia) were also purchased from Sigma-Aldrich. Xyloglucan (tamarind seed), sugar beet arabinan, barley β-glucan, wheat arabinoxylan, larch arabinogalactan, konjac glucomannan, carob galactomannan and guar bean galactomannan were purchased from Megazyme.

### Strain growth

All reagents were purchased from Sigma-Aldrich, unless otherwise stated, and were of microbiological grade. A lyophilised pellet of cultured *Chitinophaga pinensis* strain UQM 2034^T^ was purchased from DSMZ (designated DSM–2588), and propagated on LB media plates supplemented with kanamycin at 50 μg mL^−1^, to which the bacterium has innate immunity [8].

Samples (100 μl) from starter cultures (10 mL) grown in LB were initially inoculated into M9 minimal media (15 mL) containing glucose (0.5%) or an alternative carbohydrate as a carbon source (0.5%) to study growth on a range of polysaccharides [23]. Samples were taken at regular intervals and OD readings (A_600_) used as an indirect measure of cell density according to a protocol employed for other filamentous Bacteroidetes [24]. Samples of some cultures were also retained for later analysis of carbohydrate structures by HPAEC-PAD (see below). Minimal medium not supplemented with carbohydrate served as a control, and supported no growth. Cultures were incubated at 30°C with rotary shaking at 180 rpm. For subsequent analyses of proteins produced during growth, cultures of 50 mL were inoculated (three biological replicates).

### Preparation of protein fractions from cultures of *C*. *pinensis*

The total secretome of a culture was collected by centrifugation at 6,000 *g* for 15 minutes at 4°C to pellet cells. Based on initial growth curve analyses, secretomes were harvested approximately at late exponential phase; specifically, this was day 7–14, depending on the growth rate for each carbon source. For assays of secreted activity, secretomes (50 mL) were filtered (0.25 μm) (Nalgene, USA), then concentrated around 10 times, desalted and washed several times into dH_2_O using 5 kDa cut-off Amicon Ultra centrifugal filters (Millipore).

Periplasmic proteins were collected using an osmotic shock method outlined in Larsbink *et al*., 2011 [25]. Briefly, cells were washed with 10 mL of 50 mM Tris-HCl (pH 7.7) and collected by centrifugation at 4400 *g* for 10 min at 4°C; the media contained secreted proteins. The pellet was resuspended in 50 mL of 30 mM Tris-HCl, 20% (w/v) sucrose and 1 mM EDTA (pH 8.0), and the cells were incubated at room temperature for 10 min. The cells were then collected by centrifugation at 4400 *g* for 15 min at 4°C. Ice-cold 5 mM MgSO_4_ (50 mL) was added and the cells were incubated on ice for 10 min. The cells were again collected by centrifugation at 14000 *g* for 10 min at 4°C and the supernatant also retained.

The cell pellet was resuspended in 50 mM sodium phosphate buffer (pH 7.4) and sonicated to lyse cells. The lysate was centrifuged at 5000 *g* for 10 min at 4°C to remove debris. Using an ultracentrifuge, the supernatant fluid was centrifuged at 100000 *g* for 1 h at 4°C. The supernatant liquid from this round of centrifugation contained soluble proteins and was retained. The pellet, containing membrane-bound and membrane-associated proteins, was resuspended in 100 mM sodium carbonate buffer (pH 9) to remove trapped soluble proteins and/or weakly membrane-associated proteins and centrifuged again at 100000 *g* for 1 h at 4°C to obtain integral membrane proteins. The supernatant fluid from this step again contained soluble proteins and/or weakly membrane-associated proteins. The final pellet, containing membrane proteins, was resuspended in 1 mL of 50 mM sodium phosphate buffer (pH 7.4).

### Production of recombinant proteins

#### Cloning

The Cpin_5740 gene was amplified from genomic DNA by PCR and inserted into the vector pNIC28-Bsa4 by ligation independent cloning. The resulting expression construct contained a hexahistidine tag and a TEV-protease cleavage site (MHHHHHHSSGVDLGTENLYFQS) at the N-terminus. Correct in-frame insertion was confirmed by plasmid sequencing. Cloning was performed at the Karolinska Institutet/SciLifeLab Protein Science Facility (http://psf.ki.se).

#### Site-directed mutagenesis

Point mutations were introduced into the plasmid harbouring the Cpin_5740 gene by PCR amplification using the Pfx enzyme and buffer system (Life Technologies/Thermo Fisher Scientific). Table A in S1 File describes the primers used to generate each mutant plasmid. The PCR program utilised in each case was as follows: 94°C 5 minutes, 22 cycles of [94°C 30 seconds, 55°C 1 minute, 68°C 6 minutes], 68°C 15 minutes. Following Dpn1 treatment to degrade methylated parental plasmid DNA and subsequent PCR Clean-Up (Qiagen), mutated plasmids were transformed into OneShot Top10 cells (Life Technologies) by heat shock at 42°C for 30 seconds. Plasmid sequences were confirmed to contain the desired mutation by sequencing, performed by Eurofins Genomics, Germany.

#### Expression and purification

Plasmids containing the wild-type and mutant Cpin_5740 genes were transformed into *E*. *coli* BL21 (DE3) (Life Technologies) cells by heat shock at 42°C for 30 seconds. The cells were grown at 37°C with shaking in LB medium containing kanamycin (50 μg mL^−1^), to an OD_600_ of 0.4–0.6, at which point protein expression was induced by addition of 0.2 mM IPTG (isopropyl-D-galactopyranoside) and the temperature was lowered to 25°C. Protein expression continued for 2 days, after which the cells were collected by centrifugation at 4000 *g* for 10 minutes. The cells were resuspended in Buffer A (20 mM sodium phosphate pH 7.4, 500 mM sodium chloride, 20 mM imidazole) and lysed by sonication, followed by centrifugation at 17000 *g* for 30 minutes. The supernatant liquid was loaded onto 5 mL HiTrap IMAC FF columns (GE Healthcare) using an ÄKTA FPLC system (GE Healthcare Life Sciences) and washed thoroughly with Buffer A. Each protein variant was purified on a separate, unused column. His-tagged proteins were eluted using a linear gradient of 0–100% Buffer B (20 mM sodium phosphate pH 7.4, 500 mM sodium chloride, 500 mM imidazole) over typically 4 column volumes. Eluted proteins were concentrated and exchanged into 50mM sodium phosphate pH 7.4 using Amicon Ultra centrifugal filters (Millipore). Liquid chromatography electrospray ionisation MS was used to verify the correct molecular weight of purified proteins [26]. Each mutated variant of the protein was purified separately on virgin resin to avoid any cross-contamination [27].

#### Size exclusion chromatography (SEC)

An ÄKTA FPLC system was used to assess the apparent molecular mass of the Cpin_5740 gene product in solution by SEC on a Sephacryl S–300 HR (750 ml) column (GE Healthcare Life Sciences). Protein was loaded onto the column at 2 g L^−1^, with a volume of 2 mL, and eluted with 50 mM Tris–HCl buffer pH 7.0, 100 mM NaCl with a flow rate of 0.4 mL min^−1^. The void volume of the column was determined to be 102 mL using blue dextran. The molecular mass of the protein was assessed by comparing the elution volume with that of a series of standard proteins of known molecular weight in the range of 6.5 kDa to 66 kDa (Sigma Aldrich product code MWGF70).

### Enzyme activity assays

#### PNP-glycoside assays

Assays in which the PNP-glycosides d-Gal-α-PNP and l-Ara*p*-β-PNP were used as substrates were monitored for the release of 4-nitrophenolate at *A*_410_, using a Cary 50 spectrophotometer. For an initial activity screen of *C*. *pinensis* secretomes, stopped assays were performed: substrate at 2 mM was incubated with 100μl of concentrated secretome for 2 hours at 30°C in 50 mM sodium phosphate buffer, pH 7. Following incubation, an equal volume of 200 mM Na_2_CO_3_ was added to terminate the reactions by raising the pH to 11. An extinction coefficient of 18500 M^−1^ cm^−1^ was used to calculate product concentration from absorbance values [28].

A stopped assay was also used to determine the optimum pH and temperature conditions for the enzyme. Substrate (2 mM) was incubated with enzyme (125 nM) in a total reaction volume of 200 0 mine the optimum pH and temperature conditions for the enzyme. Substrate (2 mM) was incubated with formate, sodium acetate, sodium succinate, HEPES, sodium phosphate, glycyl glycine, and glycine, over a pH range from 2.5 to 10.0. The optimum pH for the wild-type enzyme was determined to be pH 5.0 (50 mM sodium citrate buffer) using the pNP-β-L-Ara*p* substrate (*vide infra*, Results and Discussion). This buffer was used to perform the same reaction at a range of temperatures, and the optimum was found to be 30°C (*vide infra*, Results and Discussion). These conditions of pH and temperature were used for all subsequent kinetic analyses of all enzyme variants acting on pNP-β-L-Ara*p*, pNP-α-D-Gal*p* and larch arabinogalactan.

For kinetic analyses of hydrolysis of PNP-glycosides by pure enzyme, a continuous assay was used in a Cary 300 spectrophotometer. A standard curve for pNP in 50 mM sodium citrate buffer, pH 5.0 gave the extinction coefficient 1415 M^-1^cm^−1^, which was used to calculate product concentration from absorbance values. The range of substrate concentrations utilised in kinetic analysis was 0–25 mM for pNP-β-l-Ara*p*, and 0–40 mM for pNP-α-d-Gal*p*. A control experiment without enzyme was performed for each rate analysis, to account for spontaneous substrate hydrolysis. Kinetic experiments were performed in 50 mM sodium citrate buffer at pH 5.0 and 30°C. All quantitative assays of enzyme activity were performed in triplicate.

#### Assay for the specific detection of arabinose release

A linked galactose dehydrogenase/galactose mutarotase assay kit (Megazyme product code E-GALMUT) was used to quantify the release of arabinose from arabinogalactan [29]. The release of arabinose led to the stoichiometric reduction of NAD^+^ to NADH, giving an increase in *A*_340_ (ε 6230 M^−1^ cm^−1^ at pH 7 [30]), which was read continuously using a Cary 300 spectrophotometer. Kinetic experiments were performed in 50 mM sodium citrate buffer at pH 5.0 and 30°C. The range of substrate concentrations used in kinetic analysis was 0–160 mg mL^−1^. A control experiment without enzyme was performed for each rate analysis, to account for spontaneous substrate hydrolysis. All quantitative assays of enzyme activity were performed in triplicate.

#### Enzyme product profiles

Reaction products were analysed on a Dionex ICS-3000 HPLC system operated by Chromeleon software version 6.80 (Dionex) using a Dionex Carbopac PA1 column. Solvent A was water, solvent B was 1 M sodium hydroxide and solvent C was 200 mM NaOH with 170 mM Na acetate. The following programme was employed: pre-wash and column calibration -14–-7 min 60% B, 40% C (1 mL min^−1^); -6–0 min 100% A (1 mL min^−1^); sample injection 0–5 min 100% A (0.5 mL min^−1^); gradient elution 5–20 min 0–30% B (0.5 mL min^−1^).

To analyse the products of polysaccharide degradation by secretomes, 1 mL assays were prepared using substrate at 1 mg mL^−1^, which was incubated with ~400 μg mL^−1^ protein for up to 4 days at 30°C in 50 mM sodium citrate buffer, pH7, prior to analysis by HPAEC-PAD. Samples (200 μl) were also taken from cultures during growth on a range of carbohydrates. These samples were boiled to stop all enzyme reactions, concentrated by lyophilisation, resuspended in 50 μl of water, and analysed by HPAEC-PAD to observe oligosaccharide production and degradation during growth. Finally, pure protein (100 nM) was incubated with polysaccharide at 1 mg mL^−1^, for 16 hours at 30°C in 50 mM sodium citrate buffer, pH 5.0, prior to analysis by HPAEC-PAD.

### Identification of the Cpin_5740 gene product in the native secretome

#### Generation and purification of antibodies specific for the Cpin_5740 gene product

For eventual use in a Western blot intended to probe for the presence of the protein, antibodies were raised in rabbits against the recombinant Cpin_5740 gene product (AgriSera AB, Vännäs, Sweden). Pre-sera from rabbits to be used in antibody generation were screened by Western blot for natural antibodies to the protein of interest, and were determined not to be reactive to the Cpin_5740 gene product. The immunisation procedure was as follows; Immunisation 1: 200 μg of antigen (1.25 mg mL^−1^) and FCA (Freund's complete adjuvant); Immunisations 2, 3 and 4 (respectively 1, 2 and 3 months later): 100 μg of antigen (1.25 mg mL^−1^) and FCI (Freund's incomplete adjuvant). The final bleed was performed 10 days after the final immunisation.

Polyclonal antibodies were purified from the final serum by affinity purification at Agrisera. Briefly, the recombinant Cpin_5740 gene product protein was first coupled to a 1 mL HiTrap NHS-activated HP Column (GE Healthcare) according to the manufacturer's instructions. The column was washed using an ÄKTA Prime system (GE Healthcare) with several column volumes of PBS pH 7.4. Two mL of 10×PBS were added to 20 mL of antiserum and this solution was applied to the recombinant protein-coupled HiTrap column. The column was washed with several column volumes of PBS. Antibodies bound to the column were eluted with 200 mM glycine in 1.0 mL fractions, into tubes containing 50 μl of 1 M Tris to neutralise the eluent. Fractions with A_280_>0.1 were pooled and precipitated with saturated ammonium sulphate overnight. The solution was then centrifuged at 5000 *g* for 15 minutes and the pellet was dissolved in 1 x PBS pH 7.4. Traces of ammonium sulphate were removed using PD10 columns from GE Healthcare. The concentration of the purified antibody was 2.7 g L^−1^.

#### Western blot analysis of the *C*. *pinensis* secretome

A Western blot analysis was performed to probe for the Cpin_5740 gene product in native *C*. *pinensis* secretomes. As described above, total secretomes were collected from 50 mL liquid cultures of *C*. *pinensis* by centrifugation to pellet cells. Secretomes were concentrated approximately 50 times and utilised in Western blots. A total of 100 μg of each secretome was loaded and run on SDS-PAGE. TBST buffer (tris-buffered saline (50 mM Tris-HCl, 150 mM NaCl, pH 7.4) with 0.1% Tween–20) was used for washing and dilutions throughout the Western blot protocol. The membrane was washed 5–6 times (~5 minutes each) between each step in the following procedure. After blotting proteins from an SDS-PAGE gel, the membrane was first blocked with a solution of TBST buffer + 3% BSA for 1 hour at room temperature to reduce non-specific binding. The purified antibodies were used as the primary antibody (1:1000 dilution, 1 hour incubation at room temperature with 1% BSA), with anti-rabbit IgG coupled to HRP (Sigma Aldrich) as the secondary antibody (1:10,000, 1 hour incubation at room temperature with 1% BSA). Final visualisation was achieved by chemiluminescence using the luminol-based Amersham ECL Western Blotting Detection Reagent (GE Healthcare) and a Fujifilm Intelligent Dark Box with LAS–1000 camera and software.

### Bioinformatics

A sequence alignment of the GH27 catalytic domains of 71 protein sequences (49 characterised and 22 uncharacterised proteins, identified by BLAST searching of GH27 enzymes with known activity and/or structure) was performed using the online Clustal Omega server [31]. Table B in S1 File provides details of the uncharacterised proteins included in the analysis. Full sequences of the catalytic domains of these proteins were used for an initial sequence alignment, which revealed the presence in *Cp*Arap27 of a number of loop insertions in conserved positions, comprising between 6 and 34 amino acids. For subsequent sequence alignments which were used to generate a phylogenetic tree, these loops were removed from the sequences, as in previous phylogenetic analyses of the family [21]. The software PhyML was used to produce a phylogenetic tree of the alignment results using the Blosum62 model of amino acid substitution, with bootstrapping of results (100 replicates) [32]. The software MEGA6 was used to view the tree [33] and Adobe Illustrator CS5 was used to produce the final figure.

## Results and Discussion

### *Chitinophaga pinensis* is capable of growth on complex polysaccharides

**Fig 1:**
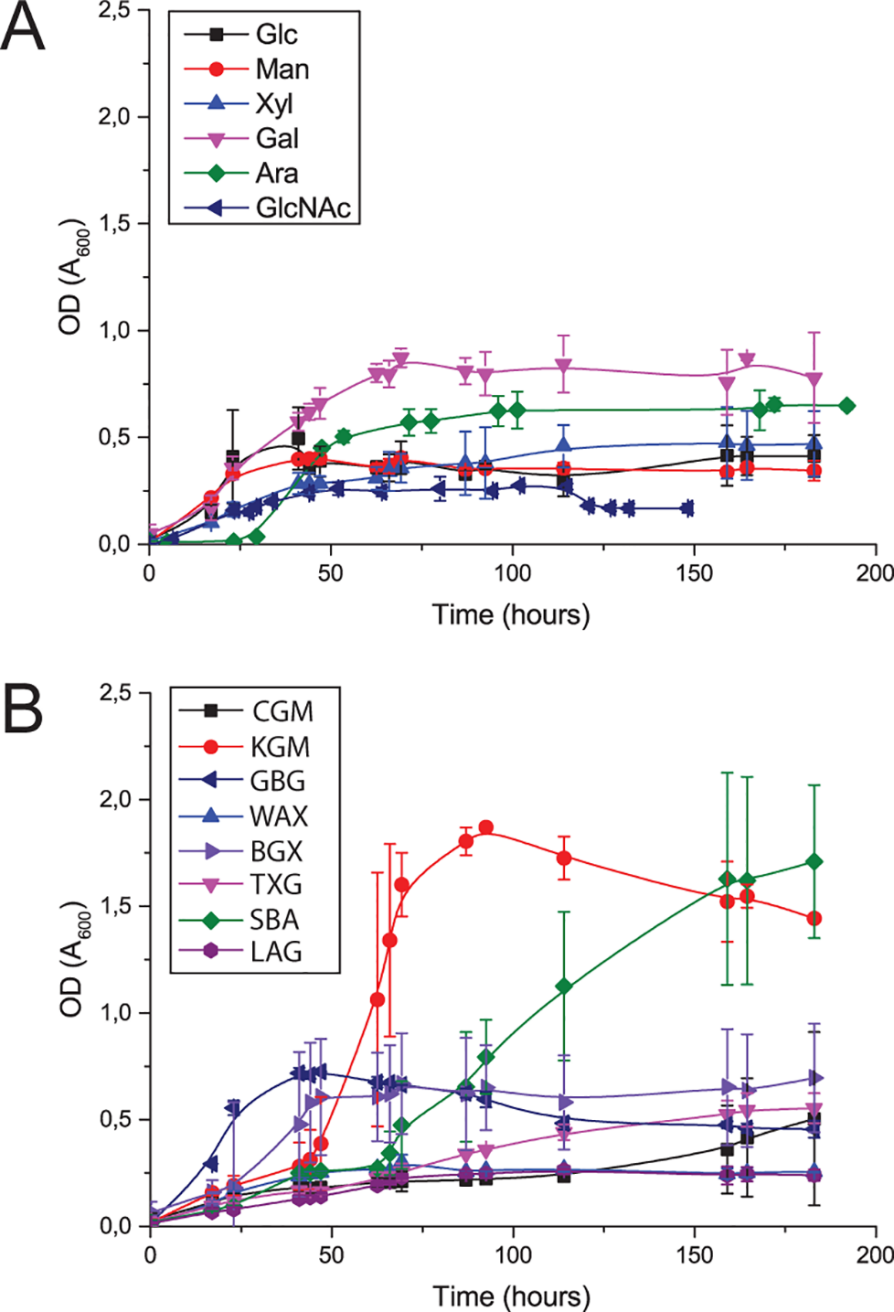
Growth curves for *C*. *pinensis* on a range of soluble carbohydrates. Growth was determined by measuring OD_600_ of samples taken at regular intervals from triplicate 15 mL cultures of *C*. *pinensis* in M9 media supplemented with various carbohydrates [24]. Growth curves for several monosaccharides (A) and polysaccharides (B) are shown. Large errors on some data points are ascribed to turbidity effects resulting from the filamentous growth habit of the bacterium; however, no visible clumps were observed during the measurement of OD_600_ values. Abbreviations for the monosaccharides are as follows: Glc, glucose; Man, mannose; Xyl, xylose; Gal, galactose; Ara, arabinose; GlcNAc, *N*-acetylglucosamine. Abbreviations for the polysaccharides are as follows: CGM: carob galactomannan. KGM: konjac glucomannan. GBG: guar bean galactomannan. WAX: wheat arabinoxylan. BGX: beechwood glucuronoxylan. TXG: tamarind xyloglucan. SBA: sugar beet arabinan. LAG: larchwood arabinogalactan.

*Chitinophaga pinensis* was screened for the ability to grow on a wide range of soluble carbohydrates of diverse structural complexity (Fig 1). In common with previous observations, growth on starch was very poor [8, 11]. As shown in Fig 1, stronger growth was sometimes observed on complex polysaccharides than on the equivalent monosaccharides. This observation has also been made for other *Bacteroidetes* species [24, 34]. Those polysaccharides supporting the greatest growth were konjac glucomannan and arabinan. Less effective growth substrates were xylans (wheat arabinoxylan and beech wood glucuronoxylan), galactomannans (from carob and guar seeds), xyloglucan and arabinogalactan. Very low levels of growth were also observed on barley β-glucan and gum arabic, but this was too weak to be accurately measured. However, it should also be noted that the liquid culture conditions may not fully reflect the native growth conditions in solid forest litter.

### β-l-arabinopyranosidase activity is secreted during growth on arabinogalactan

**Fig 2:**
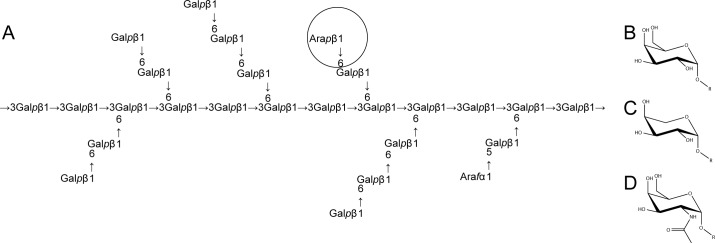
Carbohydrates known to be substrates for GH27 enzymes. (A) The complex structure of larch wood arabinogalactan comprises a backbone of β-1,3-Gal*p* residues, with β-1,6-Gal*p* decorations and side chains, as well as some terminal Ara*f* and β-L-Ara*p* (circled) substitutions. (B-D) The monosaccharide substrates of characterised GH27 enzymes are, respectively, α-D-Gal*p*, β-L-Ara*p*, and *N*-acetylgalactosamine. The complexities of carbohydrate nomenclature obfuscate the fact that β-l-Ara*p* and α-d-Gal*p* differ only in the absence or presence, respectively, of a hydroxymethyl group on the C5 position of the sugar ring [48, 49].

Sparked by an interest in galactomannan utilisation, we initially identified locus Cpin_5740, the sole GH27-encoding gene in *C*. *pinensis*, as an enzyme of interest due to the predominance of α-galactosidases in this GH family. However, recombinant expression of Cpin_5740 in *E*. *coli* subsequently revealed this enzyme to be a strict β-l-arabinopyranosidase (*vide infra*). We therefore expanded our initial screen to focus on carbohydrates containing the β-l-Ara*p* structure. There is evidence that β-linked l-Ara*p* residues are found in Type I and Type II arabinogalactans, although they seem to be relatively rare compared to l-Ara*f* residues [35–37] (Fig 2). Other polysaccharides which may contain small amounts of β-l-Ara*p* residues are gum arabic and sugar beet arabinan [38–42]. Therefore, the ability of *C*. *pinensis* to grow on the branched arabinose-containing polysaccharides larch arabinogalactan [35–37, 43], sugar beet arabinan [40, 44, 45], and gum arabic [46, 47], as well as their component monosaccharides, was analysed in more detail. Growth on the simple substrate glucose was also analysed, as a control experiment. Despite a comparable doubling rate during the exponential phase, growth on arabinogalactan was poor compared to growth on the constituent monosaccharides arabinose and galactose, with a longer lag phase and lower final OD (Fig 1). The bacterium was able to grow only very slightly on the structurally similar gum arabic, which may reflect a paucity of hydrolytic enzymes for this polysaccharide, including the apparent inability of the Cpin_5740 gene product to release monosaccharides from it (*vide infra*). Growth on sugar beet arabinan was strong and conformed more closely to the aforementioned pattern observed for other *Bacteroidetes* species where growth on polysaccharide is stronger than growth on simpler carbohydrates [24, 34]. During growth on arabinogalactan, the medium was sampled regularly for analysis by HPAEC-PAD, which showed no *endo*-hydrolysis of the polysaccharide.

**Fig 3:**
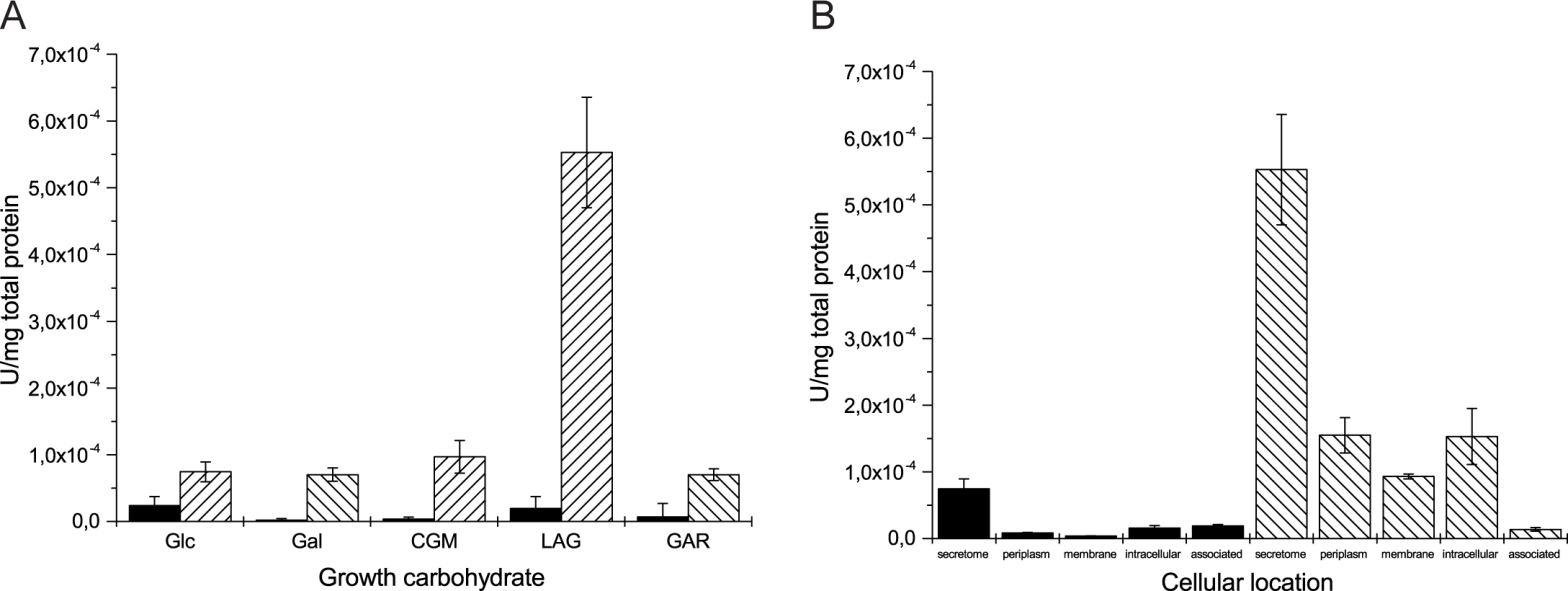
Growth on arabinogalactan induces the secretion of pNP-β-l-Arabinopyranosidase activity. (A) Hydrolysis of pNP-α-d-Gal*p* (solid) and pNP-β-l-Ara*p* (striped) by secretomes induced by different growth carbohydrates. (B) pNP-β-l-arabinopyranosidase activity is more strongly enriched in the secreted protein fraction than other cellular locations, and is much more significant in cultures induced by arabinogalactan (striped) compared to cultures induced by glucose (solid). The term ‘associated’ denotes proteins which are weakly associated with cell membranes. Abbreviations are as follows: Glc, glucose; Gal, galactose; CGM, carob galactomannan; LAG, larch wood arabinogalactan; GAR, gum arabic.

To further probe the behaviour of the bacterium under these conditions, proteins produced during growth on galactose-containing polysaccharides were tested for hydrolysis of the chromogenic *exo*-glycosidase substrates pNP-β-l-Ara*p* and pNP-α-d-Gal*p*, which revealed a significant induction of β-L-arabinopyranosidase activity during growth on arabinogalactan (Fig 3A). Moreover, this activity was predominantly localised to the secretome, versus the periplasm, cytosol, and cellular membranes (Fig 3B). Incubation of cell-free secretomes from glucose-grown control cultures and arabinogalactan-grown cultures with arabinogalactan as an assay substrate revealed that only the latter was able to release a very small amount of arabinose from the polysaccharide, in which the β-l-Ara*p* structure is quite rare [35–37].

### Locus Cpin_5740 encodes a secreted β-L-arabinopyranosidase induced by arabinogalactan

GH27 is a family of retaining enzymes notably containing α-d-galactosidases and some α-*N*-acetylgalactosaminidases [12], which recently has seen an expansion of its catalytic repertoire to include β-l-arabinopyranosidases [41, 42, 50, 51]. Locus Cpin_5740 encodes the sole member of GH27 in the *C*. *pinensis* genome [11], which we predicted to be an extracellular protein due to the presence of an SpI signal peptide [52]. We therefore identified this gene as a likely candidate to encode the extracellular β-l-arabinopyranosidase activity identified in the secretome analysis. Cpin_5740 is located on the chromosome adjacent to a gene (Cpin_5739) encoding a GH51 member, a predicted α-l-arabinofuranosidase that may also target arabinogalactan (Fig 2). Indeed, independent assays also detected hydrolysis of pNP-α- l-Ara*f* in the arabinogalactan-induced secretome (data not shown). No other predicted CAZyme-encoding genes are located nearby, but the *C*. *pinensis* genome does encode other enzymes likely to be involved in degradation of arabinogalactan, including potential β-galactosidases (members of families GH1, 2, 16, 35, and 43), α-l-arabinofuranosidases (families GH2, 43 and 51), and galactanases (families GH5, 16, 30, 35 and 53) [11, 12]. In this context, it is interesting to note that *C*. *pinensis*, a member of the phylum Bacteroidetes, does not appear to co-locate carbohydrate-active enzymes and carbohydrate-binding proteins into Polysaccharide Utilisation Loci common in *Bacteroides* species and some other Bacteroidetes [53–55].

**Fig 4:**
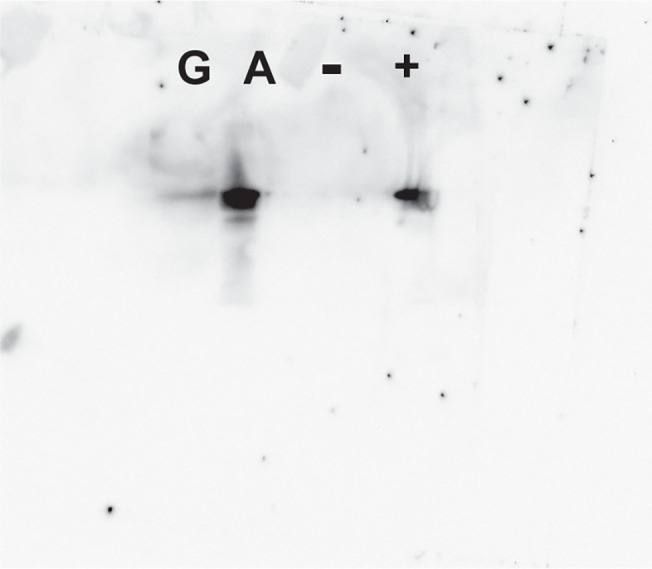
Western blot evidence that *Cp*Arap27 is secreted during growth on arabinogalactan. Western blot analysis of *C*. *pinensis* secretomes induced by growth on glucose and arabinogalactan. A band is visible which corresponds to the ~48 kDa *Cp*Arap27 protein in the arabinogalactan secretome. Protein samples on the blot are labelled as follows; G, glucose-induced secretome; A, arabinogalactan-induced secretome; -, BSA (negative control); +, pure recombinant His_6_-tagged *Cp*Arap27 (positive control). The PageRuler Plus protein ladder (Life technologies) was used to assess that the visible band was of the correct size. A total of 100 μg of protein was loaded for each of the secretomes.

The cloned Cpin_5740 gene expressed well in *E*. *coli*, typically yielding 10–30 mg protein from a 1 L culture. The hexahistidine-tagged, recombinant protein (henceforth referred to as *Cp*Arap27) was readily purified using immobilised metal affinity chromatography (IMAC) (Fig A in S1 File). Analysis by size-exclusion chromatography (SEC) indicated that *Cp*Arap27 is monomeric in solution (Fig B in S1 File). Western blot analysis using rabbit antibodies raised against this recombinant protein confirmed that secretion of *Cp*Arap27 is indeed induced during growth on arabinogalactan but not glucose (Fig 4).

The purified recombinant enzyme was subjected to an activity screen on artificial, chromogenic pNP substrates (see full list in Experimental Procedures) and was found to be strictly specific for pNP-β-l-Ara*p* (Table 1, full *v*_o_ vs. [S] plots are given in Fig D, S1 File). Using pNP-β-l-Ara*p*, optimum conditions of pH and temperature for the enzyme were determined to be pH 5.0 and 30°C (Fig D, S1 File). The observation of strict pNP-β-l-Ara*p* activity contrasts with other characterised β-l-arabinopyranosidases, which have shown low activity against the structurally similar pNP-α-d-Gal*p* (Table 2) [41, 42, 51, 56, 57]. HPAEC-PAD analysis demonstrated that *Cp*Arap27 is able to release arabinose from larch arabinogalactan as the sole reaction product (Fig C, S1 File). Using a linked assay to measure arabinose release, kinetic analysis of this reaction was performed for wild-type *Cp*Arap27 (Table 1, and Fig D, S1 File). The high *K*_*m*_ for this reaction, which is derived from the polysaccharide concentrations in the assays, likely reflects the low abundance of the target structure in arabinogalactan [35, 58, 59] (Fig 2). No arabinose release was detected when *Cp*Arap27 was assayed against the arabinose-containing polysaccharides sugar beet arabinan and gum arabic, either because the β-l-Ara*p* structure was of too low abundance or sterically inaccessible to *Cp*Arap27 in these substrates.

**Table 1:** Kinetic parameters for wild-type and mutant forms of *Cp*Arap27 against artificial and natural substrates.

	pNP-β-l-arabinopyranoside			pNP-α-d-galactopyranoside			Larch wood arabinogalactan		
Enzyme	*k*_*cat*_ (min^−1^)	*K*_*m*_ (mM)	*k*_*cat*_ / *K*_*m*_	*k*_*cat*_ (min^−1^)	*K*_*m*_ (mM)	*k*_*cat*_ / *K*_*m*_	*k*_*cat*_ (min^−1^)	*K*_*m*_ (mg mL^−1^)	*k*_*cat*_ / *K*_*m*_
Wild-type	1940 ± 215	12.9 ± 3.27	150	NA			1580 ± 135	18.0 ± 4.46	87.5
I56D	0.431 ± 0.0465	8.63 ± 2.14	0.0499	0.0124 ± 2.52 x 10^−3^	35.3 ± 11.9	3.51 x 10^−4^	160.0 ± 20.6	36.0 ± 12.9	4.44
I56E	31.7 ± 6.88	6.56 ± 3.39	4.83	6.31 ± 3.11	35.6 ± 15.2	0.177	7.95 ± 1.11	21.6 ± 8.56	0.368
I56C	24.2 ± 7.14	21.5 ± 10.4	1.13	ND^a^	ND	2.45 ± 0.2	NA^b^		
I56A	ND	ND	0.372 ± 0.0561	ND	ND	0.539 ± 0.0176	NA		
D187A	ND	ND	2.35 x 10^−3^ ± 5.18 x 10^−4^	NA			NA		
D242A	ND	ND	5.44 x 10^−3^ ± 3.78 x 10^−4^	NA			NA		

Kinetic parameters are provided for wild-type and variant forms of *Cp*Arap27 acting on three different substrates. Throughout, values are given to 3 significant figures, with standard errors of means also provided.

^a^ ND indicates a kinetic parameter which could not be determined. For reactions for which individual *k*_*cat*_ and *K*_*m*_ values are not given, values for *k*_*cat*_ / *K*_*m*_ were determined from linear curve fitting of initial rate data, at low substrate concentrations (Fig D, S1 File). Otherwise, values for *k*_*cat*_ and *K*_*m*_ were determined using Origin 9.1.

^b^ NA denotes that no activity against a particular substrate was detected.

**Table 2:** Summary of specificities of several characterised GH27 enzymes, and motivation for the mutations of *Cp*Arap27.

*Cp*Arap27 variant form		Equivalent previously characterised enzyme		
Enzyme	Ara:Gal ratio ^a^	Enzyme	*k*_*cat*_ / *K*_*m*_	Ara:Gal ratio ^a^
Wild-type	Ara only	*Gs*Abp	Ara:495 [42]	Ara (trace pNP-α-D-Gal*p*, pNP-α-L-Ara*f*)
I56E	Ara only	*Sa*Arap27A	Ara:88.1 Gal:0.5 [41]	196:1
I56D	142:1	*Sc*aGal	Gal:63.6 [21]	Gal only
I56D	142:1	*Sa*Arap27A E99D	Ara:1.5 Gal:6.7 [41]	1:4.4
I56C	1:2.2	*Fo*Ap1	Ara:9.2 Gal: 9.9 [50]	1.1:1
I56C	1:2.2	*Fo*Ap2	Ara:8.1 Gal:0.6 [50]	13.6:1
I56A	1:1.4	*No examples*	-	-

Kinetic parameters for several GH27 enzymes, with different substrate specificities and different amino acids in a key active site position, are compared. Kinetic parameters for all forms of *Cp*Arap27 are given in Table 1. The Ara:Gal ratios were determined by dividing *k*_*cat*_ / *K*_*m*_ values and normalising the smaller value to 1.

^a^ The Ara:Gal ratio of each enzyme or enzyme variant compares *k*_*cat*_ / *K*_*m*_ for hydrolysis of pNP-β-l-Ara*p* and pNP-α-d-Gal*p* respectively.

### Structural determinants of specificity in family GH27

**Fig 5:**
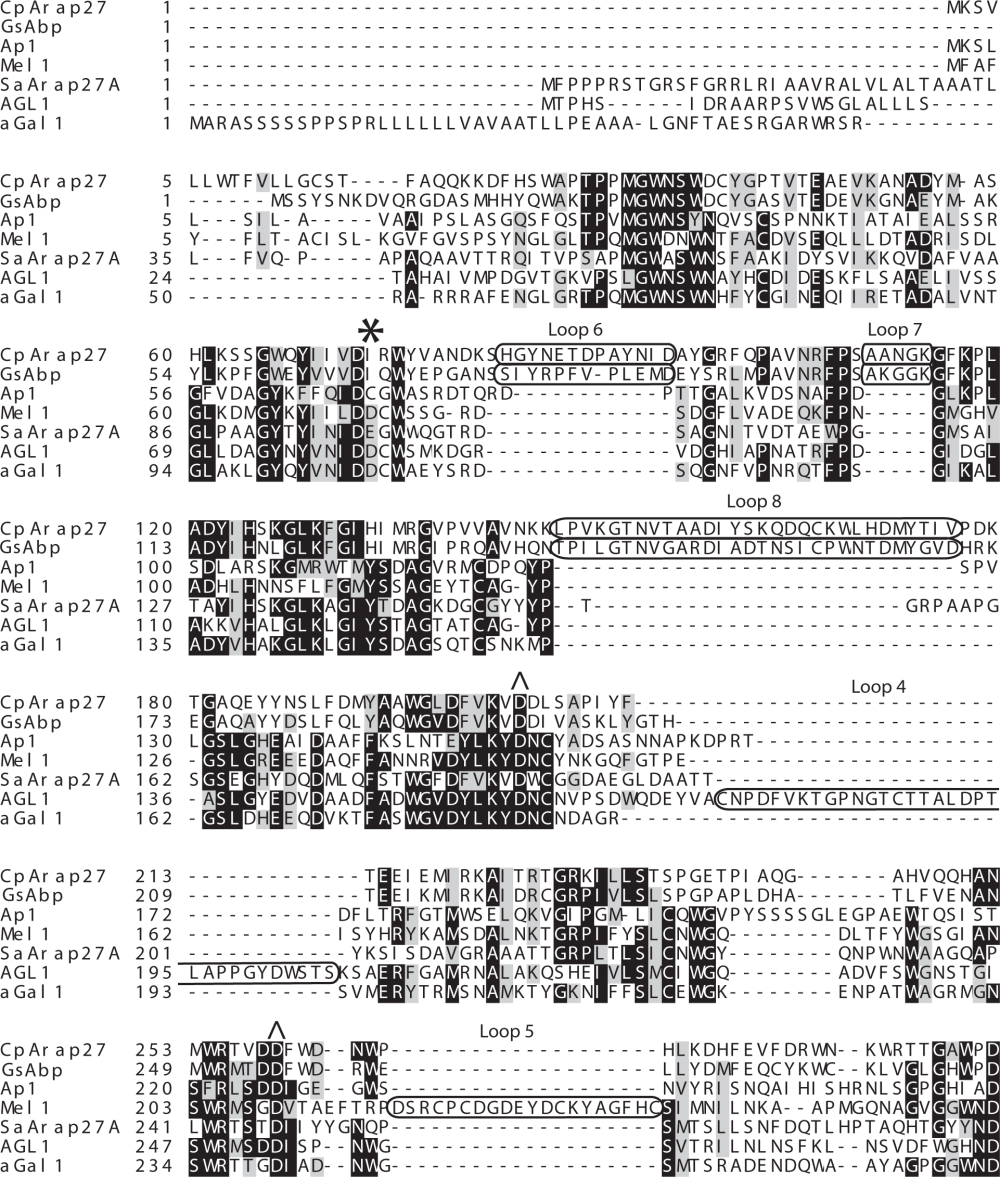
Sequence alignment of key variable GH27 regions. Sequence alignment of *Cp*Arap27 (*C*. *pinensis)*, *Gs*Abp (*G*. *stearothermophilus)* [51], Ap1 (*F*. *oxysporium*) [50], Mel 1 (*S*. *cerevisiae*) [21], *Sa*Arap27A (*S*. *avermitilis)* [41], AGL1 (*T*. *reesei*) [60], and αGal1 (*O*. *sativa*) [61]. This alignment highlights variation of amino acid in a key position for specificity (indicated with an asterisk), and shows significant loop insertions in different GH27 enzymes, highlighted in boxes. Catalytic amino acids are indicated by a caret.

A key question regarding *Cp*Arap27 in the context of GH27 is which active site features of the enzyme determine specificity for the β-l-Ara*p* substrate over the similar α-D-Gal*p* substrate. To explore this, we performed sequence alignments with previously characterised GH27 enzymes with differing substrate specificities, and subsequently produced a homology model of *Cp*Arap27 (*vide infra*). As the sequence alignment in Fig 5 shows, likely candidates for the two catalytic Asp residues of *Cp*Arap27 were identified as Asp187 and Asp242. Individual site-directed alanine mutants of these residues had drastically reduced activity against pNP substrates compared to the wild-type enzyme (Table 1, and Fig D, S1 File) [27].

*Geobacillus stearothermophilus* Abp, another GH27 enzyme, was recently described with the ability to hydrolyse pNP-β-l-Ara*p* and remove arabinose from arabinogalactan, with limited activities against pNP-α-d-Gal*p* and pNP-α-l-Ara*f* [42]. The importance of the residue Ile67 in this enzyme has recently been demonstrated by structural determination and site-directed mutation; a crystal structure of *Gs*Abp in complex with l-Ara suggests that a steric clash would occur between Ile67 and galactopyranosides in the active site pocket [51]. Sequence analysis (Fig 5) shows that *Cp*Arap27 possesses a homologous isoleucine (Ile56), a series of loop insertions, and an overall 53% sequence identity vis-à-vis *Gs*Abp. A homology model of the *Cp*Arap27 structure was generated by the Phyre2 server, using the *Gs*Abp crystal structure as a template [51, 62]. In light of the high primary structural similarity between the template and the modelled protein, a high level of tertiary structural similarity was correspondingly observed.

**Fig 6:**
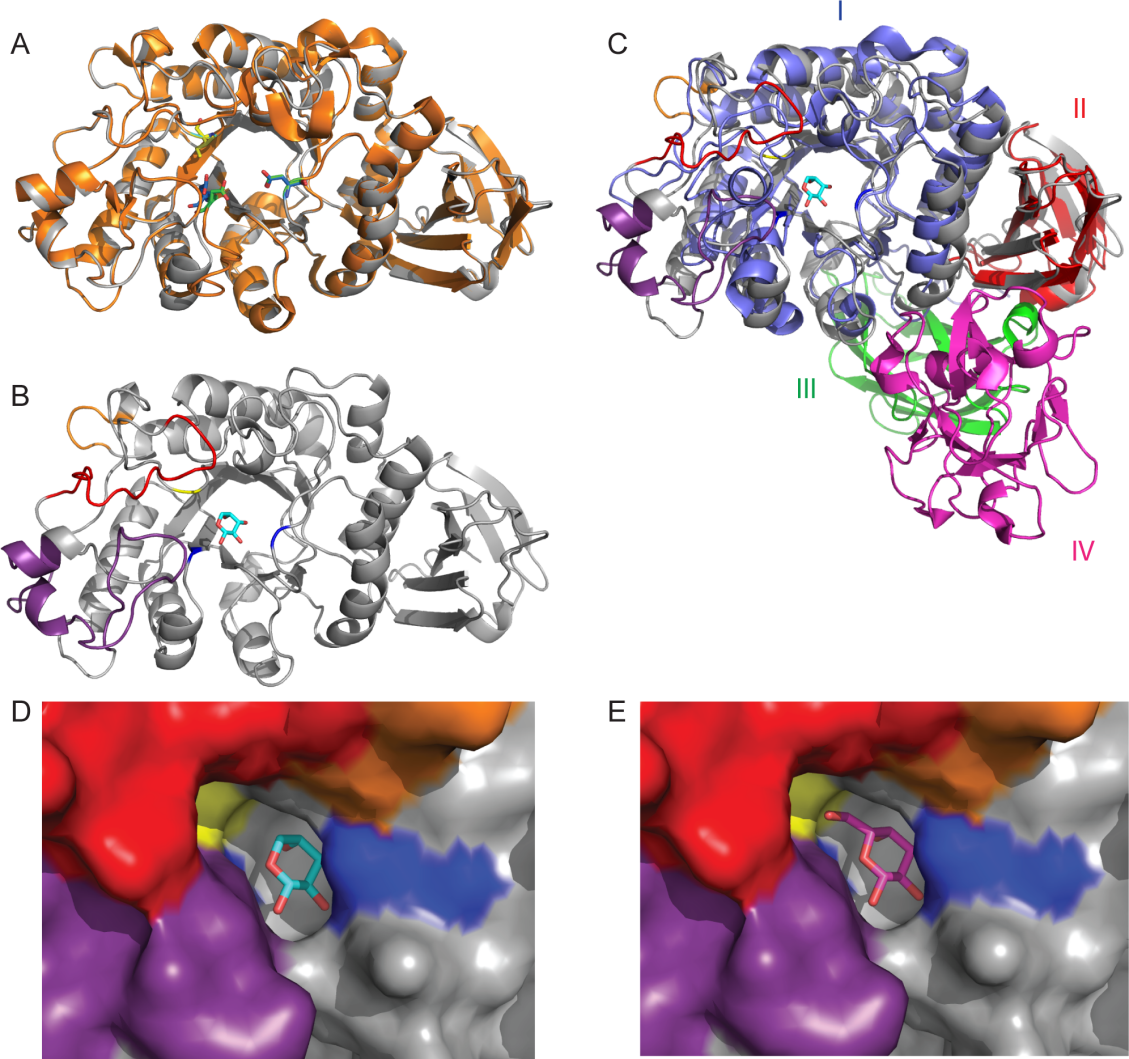
Structural comparison of *Cp*Arap27 with *Gs*Abp and *Sa*Arap27A. (A) Cartoon representation of the *Cp*Arap27 homology model (grey) and the crystal structure of *Gs*Abp (orange). Catalytic amino acids for the enzymes are picked out respectively in blue/green, while the key Isoleucine specificity determinant is yellow/cyan. (B) Secondary structural representation of *Cp*Arap27, with loop insertions highlighted as follows: L6 is red, L7 is orange, and L8 is purple. Ile56 is shown in stick form and highlighted in yellow. Catalytic amino acids are highlighted in blue. (C) Comparison of the modular structures of *Sa*Arap27A (Domains I-IV) and the homology model of *Cp*Arap27 (Domains I and II). *Sa*Arap27A is shown in 4 colours to highlight the 4 domains of this protein. *Cp*Arap27 is coloured as in panel B. A molecule of l-Ara is shown in the active site, taken from the structure of *Sa*Arap27A. (D) and (E) Surface representations of the active site of the *Cp*Arap27 homology model overlaid with, respectively, l-Ara and d-Gal from the *Sa*Arap27A structures. Catalytic amino acids are shown in blue, Ile56 is shown in yellow, and the loop insertions are again shown in purple, red and orange. The Isoleucine shown here is predicted to hydrophobically clash with a hypothetical d-Gal substrate, as shown for *Gs*Abp [51].

Superimposing the *Cp*Arap27 model structure with l-Ara and d-Gal from ligand-bound structures of *Sa*Arap27A (the first GH27 shown to possess β-l-arabinopyranosidase activity) suggests that the side chain of Ile56 in *Cp*Arap27 would bias specificity towards arabinopyranose in the same manner as Ile67 in *Gs*Abp does (Fig 6D and 6E). The model also indicates that the loop insertions in *Cp*Arap27 and *Gs*Abp, identified in sequence alignments (Fig 5) and not present in *Sa*Arap27A, may be significant for activity, as they contribute to the architecture of the active site pocket.

In characterised GH27 enzymes, the amino acid position corresponding to Ile56 contains an Asp in α-d-galactopyranosidases [21], a Glu or Ile in β-l-arabinopyranosidases [41, 42], and a Cys in two catalytically flexible fungal enzymes, *Fo*Ap1 and *Fo*Ap2 [50]. Due to the apparent correlation between this residue and enzyme specificity (Table 2), we were intrigued by the possibility of engineering *Cp*Arap27 to reflect the other specificities displayed by members of family GH27. Ile56 was mutated to each of these alternate amino acids and the specificity and kinetic parameters of these variants were explored (Table 1, and Fig D, S1 File)

As predicted, mimicking the active site of typical GH27 α-d-galactosidases by generating the I56D variant form of *Cp*Arap27 did introduce hydrolytic activity toward pNP-α-d-Gal*p* (Table 1), as was previously demonstrated for *Gs*Abp [51] (Table 2). Similarly, the I56E variant showed some catalytic promiscuity (Table 1), as has been observed for *Sa*Arap27A [41] (Table 2). Both of these variants were catalytically feeble and in neither case was hydrolysis of the galactopyranoside substrate the most significant activity. For *Cp*Arap27 I56D, which has the Asp typical of GH27 α-d-galactosidases, and *Cp*Arap27 I56E, comparison of the *k*_*cat*_/*K*_*m*_ values for hydrolysis of pNP-β-l-Ara*p* and pNP-α-d-Gal*p* indiciates Ara*p*:Gal*p* preferences of 12:1 and 39:1 (Ara*p*:Gal*p* for the wild-type enzyme = 1:0), respectively. The *Cp*Arap27 I56E mutant is a mimic of the wild-type *Sa*Arap27A (Ara:Gal ratio 67:1) and has similar levels of preference for the l-arabinopyranosyl substrate, but is catalytically much weaker [41]. Similarly, the I67D mutation of *Gs*Abp induced a 3-fold increase in hydrolysis of pNP-α-d-Gal*p*, as well as a 2.7-fold decrease in hydrolysis of pNP-β-l-Ara*p* [51]. Further, although *Sa*Arap27A was previously modified to prefer the galactoside by mutation of the key Glu to an Asp (Table 2), the resulting E99D mutant (Ara:Gal ratio 1:9) was also catalytically enfeebled in the wild-type activity, similar to the results obtained here for the *Cp*Arap27 I56D variant (Tables 1 & 2, and Fig D, S1 File). Notably, the *Cp*Arap27 I56A and I56C variants are able to hydrolyse both pNP-α-d-Gal*p* and pNP-β-l-Ara*p* with roughly equal efficiency, but are nonetheless also poor catalysts (Table 1).

In all cases, despite alterations in the activities toward the artificial chromogenic glycosides, no gain-of-function for hydrolysis of arabinogalactan was observed for any of the *Cp*Arap27 variants. For the I56D and I56E variants, arabinose could be identified as the sole hydrolysis product of larch arabinogalactan, as was observed for the wild-type enzyme (Fig C, S1 File). Despite prolonged incubation with high enzyme concentration, no reaction products were detected by HPAEC-PAD for *Cp*Arap27 I56A, I56C or the catalytically inactive D187A and D242A. Neither the wild-type nor any of the mutant enzymes showed any activity on sugar beet arabinan, gum arabic, linear galactan or galactomannan polysaccharides.

Although all of the variants examined do have some ability to bind and hydrolyse the chromogenic galactopyranoside, it is clear that key enzyme-substrate interactions that enable efficient hydrolysis in natural GH27 α-d-galactopyranosidases still have not been fully accounted. As mentioned above, *Cp*Arap27 and *Gs*Abp also share other important structural features, including several inserted loop regions which are not present in *Sa*Arap27A or in α-d-galactosidases of the family. Loop insertions into the general (α/β)_8_ barrel fold are known to significantly affect the substrate specificity and oligomerisation of GH27 enzymes [21, 22], and indeed all TIM-barrel containing GHs [63, 64]. Furthermore, past attempts to engineer the specificity of non-glycosidase TIM-barrel containing enzymes via loop exchange have been more successful than simple mutagenic alterations [65, 66]. To provide a broader view of structural variations within GH27 and their impact on substrate specificity, we performed a detailed phylogenetic analysis including all functionally and structurally characterised members of the family.

### Phylogenetic analysis of GH27

**Fig 7:**
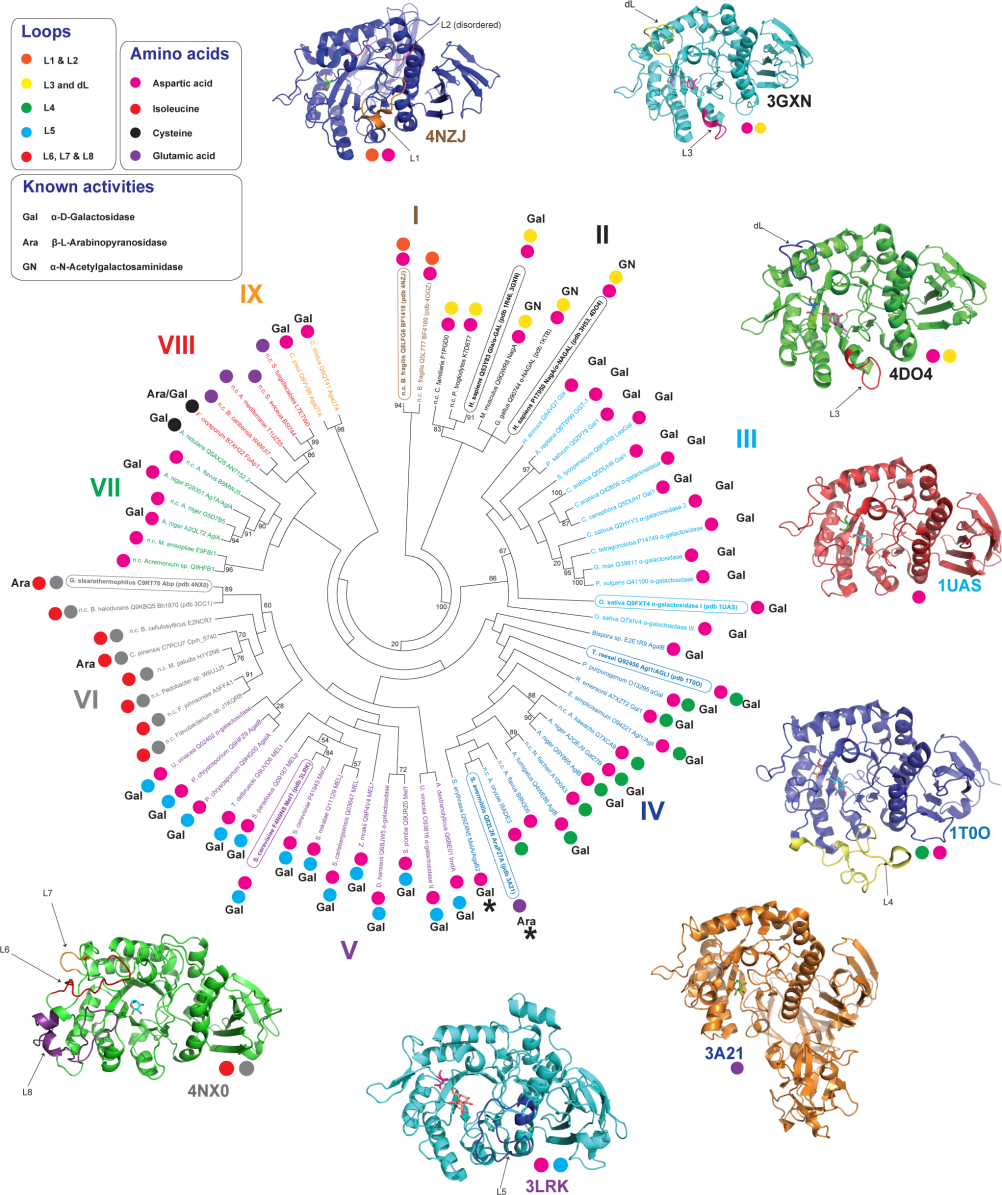
Phylogenetic analysis of family GH27. Proteins are labelled with organism name and *UNIPROT* code. The phylogenetic tree includes all characterised GH27 enzymes identified as such on the CAZy database at the time of writing, plus several as yet uncharacterised gene products, identified by sequence homology to characterised enzymes using pBLAST analysis. Proteins for which a 3D structure is available are shown in bold and pdb codes are provided. Examples are shown in cartoon form around the tree to highlight conserved structural elements within a clade, and the name of an illustrated protein is circled on the tree. Each clade is labelled in a specific colour. Characterised enzymes are labelled with Ara or Gal to indicate their preferred substrate. Currently uncharacterised proteins are indicated with n.c.. Finally, proteins not conforming to the pattern of loop insertions or active site amino acids otherwise well conserved for their clade are highlighted with an asterisk. The phylogenetic tree was produced using a Clustal Omega alignment [31] and the PhyML software [32], and the tree was visualised using MEGA5 [67].

A phylogenetic analysis of GH27 presented by Fernández-Leiro *et al* in 2010 showed the importance of loop insertions in controlling protein oligomerisation and enzyme specificity, including the preference of α-galactosidases for the terminal or inner galactosyl side-chains of a polysaccharide [21]. A significant increase in knowledge of the specificities of GH27 enzymes, in particular the recent revelation of β-l-arabinopyranosidase activity in the family, warranted an update to this phylogeny, which is presented in Fig 7. Our analysis reveals several new clades which share distinct patterns of loop insertions and specificity-determining amino acid residues, and shows again that there is a strong correlation between specificity and the presence of specific loops. Specifically, loop insertions possessed by members of the group to which *Cp*Arap27 belongs were not identified by previous phylogenetic analyses [21, 22]. From this new tree it is clear that certain subsets of GH27 enzymes are very well studied, while other groups still require investigation in order to better understand the full complexity of this enzyme family. The groups identified by this analysis, described individually below, may have application in predicting the activity of GH27 enzymes yet to be characterised.

The first apparent group, Group I, comprises only two proteins, which are closely related enzymes from *Bacteroides fragilis*. Neither of these has been biochemically characterised so no predictive conclusions can be drawn from this group, although crystal structures are available for both (unpublished). These proteins have an Asp at the critical position described above, and two loop insertions (L1 & L2) of currently unknown influence on specificity, but which appear not to contribute directly to the active sites.

Group II comprises mostly mammalian enzymes and includes examples of both α-galactosidases [22] and α-*N*-acetylgalactosaminidases [68]. These enzymes have very highly conserved active sites with an Asp in the specificity-influencing position (Table 2), and as was previously noted [21, 22], all include a loop (L3) which is a major specificity determinant. This loop has previously been referred to as the “2 position recognition loop”, and the presence of a very short insertion in this region causes a structural rearrangement that allows the active site to preferentially accommodate the bulkier GalNAc residue over a Gal residue [22, 69, 70]. These enzymes also include a conserved loop with amino acids important in dimerisation (dL). The reader is referred to the insightful work of Garman and Garboczi for a detailed discussion of enzyme structure-function relationships in this clade [22].

Groups III and IV comprise α-Galactosidases (with one important exception in Group IV), but differ in the specificity of the enzymes for targeting the branching galactosyl residues on substrates such as galactomannan. All enzymes have an Asp in the key position mentioned above. The loop insertion L4 in most members of Group IV may contribute to the specificity of these enzymes for polysaccharides with galactosyl branches along their length [60], while members of Group III, which lack this loop, mostly hydrolyse galactose branches on terminal backbone residues of polysaccharides, although some are flexible in this specificity. All of the enzymes in these groups appear to be monomeric, lacking the loop insertions necessary for oligomerisation, as exemplified by the structure of the catalytically flexible Group III rice (*Oryza sativa*) α-galactosidase [70].

It should be noted here that Group IV also includes the first characterised β-L-arabinopyranosidase, *Sa*Arap27A [41]. This enzyme is a significant outlier in this group, lacking the very highly conserved L4 insertion, and possessing a Glu rather than an Asp at the critical position. Further, *Sa*Arap27A possesses two additional domains (domains III and IV shown in Fig 6) which are not present in the other members of Group IV, or in other β-l-arabinopyranosidases. Domain III, which has a β-jellyroll conformation, makes contact directly with the enzyme active site in domain I, while the C-terminal domain IV of *Sa*Arap27A is a family 13 carbohydrate-binding module (CBM13) that may mediate association to large polysaccharide substrates such as arabinogalactan [41]. These extensive structural modifications, plus the presence of the Glu in the conserved active site–adjacent position, may explain the different specificity of *Sa*Arap27A compared to the other members of Group IV, although the manner in which these significant differences arose is currently unclear in the absence of a larger number of characterised examples. A BLAST search against the non-redundant protein database indicates that the domain architecture of this enzyme is common to predicted α-galactosidases from *Streptomyces* species, many of which contain the active site Glu, indicating that they may also be β-l-arabinopyranosidases.

Group V includes several characterised α-galactosidases. Enzymes in this group have an Asp in the critical position, and contain L5, a loop insertion identified previously as having involvement in protein oligomerisation and in the creation of binding sites [21]. This loop is the structural determinant which restricts the access of branching galactosyl residues to the active site, causing an enzyme to be specific for terminal galactosyl residues [21].

With respect to previous phylogenetic analyses [21, 22], Group VI is a newly apparent clade that includes two structural representatives (*Gs*Abp and Bh1870) and two characterised β-l-arabinopyranosidases (*Cp*Arap27 and *Gs*Abp). The presence in these enzymes of an Ile at the key position, which engenders specificity for l-arabinosyl substrates over d-galactosyl structures ([42, 51] and the present work), distinguishes the members of this group from other GH27s. Further, all members of this group possess the inserted loops L6, L7 & L8, with L6 and L7 contributing directly to the active site architecture. L8, in particular, is found at the dimerisation interface of *Gs*Abp [51]. Interestingly, whereas *Gs*Abp has been shown by SEC to be a tetramer in solution, and by crystallography to comprise a ‘dimer of dimers’, our analysis indicates that *Cp*Arap27 is monomeric in solution (Fig B in S1 File). The amino acid sequence of L8 similar, but not identical between these two proteins (Fig 5), and analysis of the structure of the *Gs*Abp tetramer suggest that specific amino acids in this loop mediate dimerisation, which rationalises this apparent discrepancy in oligomerisation behaviour [51].

The remaining three groups have relatively few members, and possess no distinguishing loop insertions into the overall protein fold. Groups VII and VIII are broadly distinguished by the presence of Asp (Group VII) or Glu (Group VII) in the key position, with certain exceptions. An *Aspergillus nidulans* [71] galactosidase of Group VII has a Cys residue in this position. Likewise, the only characterised member of Group VIII is an enzyme annotated as a bifunctional α-d-galactosidase/β-l-arabinopyranosidase (*Fo*Ap1) which also has a Cys in this position; this enzyme is able to cleave both substrates [50] but has a preference for galactose. In light of the variation between Asp and Cys in members of these groups, prediction of enzyme specificity by extrapolation from these examples is limited. Likewise, the implications of a Cys in an important, specificity-determining position in members of Groups VII and VIII, which appear to be otherwise highly similar in the key structural elements discussed here, is currently unclear. Finally, Group IX is a budding clade of α-galactosidases which possess an Asp in the key position. The two enzymes represented in this group are both active on galactomannan [61, 72].

In summary, our phylogenetic analysis highlights how the (α/β)_8_ barrel fold of GH27 enzymes has been modified in nature to incorporate specific active site residues and loop insertions which affect specificity and oligomerisation. The presence or absence of many of these features may help guide functional prediction and provides a framework for the characterisation of novel GH27 members.

## Conclusions

*C*. *pinensis* is a free-living, saprophytic bacterium with a significant capacity to secrete diverse glycoside hydrolases for the utilisation of complex polysaccharides for growth. Among these is the GH27 enzyme *Cp*Arap27, which is highly specific for the hydrolysis of β-l-arabinopyranoside substrates. Sequence alignment and phylogenetic analysis have demonstrated that a key amino acid position in the family influences specificity in GH27 enzymes, with individual members having absolute specificity for β-l-Ara*p*, increasing levels of activity towards α-D-galactopyranose, or absolute specificity for α-d-Gal*p* (Fig 7, Table 2). However, we and others have shown that manipulation of the specificity of these enzymes by site-directed mutagenesis of this amino acid is inevitably accompanied by a significant penalty to catalysis. A strictly reductionist approach to specificity engineering is therefore clearly limited in the GH27 system, which indicates that other structural features make important contributions to specificity. Indeed, the β-l-arabinopyranosidases of this family are distinguished not just by the presence of an important Isoleucine in the active site, but also by major loop insertions, which are unique to the phylogenetic clade in which they are found. Nonetheless, the phylogeny presented here serves as a useful guide for predicting further β-l-arabinopyranosidases in GH27, thereby informing future bioinformatics and enzyme structure-function studies.

## Supporting Information

S1 File Supporting information. Tables A-B and Figures A-D.
(PDF)
