# Supplementary material for: Growth of Chitinophaga pinensis on Plant Cell Wall Glycans and Characterisation of a Glycoside Hydrolase Family 27 β-l-Arabinopyranosidase Implicated in Arabinogalactan Utilisation
Source: PLoS One. 2015 Oct 8;10(10):e0139932. doi: 10.1371/journal.pone.0139932 (PMC4598101; doi:10.1371/journal.pone.0139932)
Supplement: S1 File — Tables A-B and Figures A-D. (PDF) [file pone.0139932.s001.pdf]

S1. Supporting information

Supporting tables

Table A. Primers used to produce mutant forms of the Cpin_5740 gene.

Mutation	Forward primer	Reverse primer
I56A	tggcaatacattatagtagacGCAcgctggtatgtggccaatg	cattggccacataccagcgTGCgtctactataatgtattgcca
I56C	tggcaatacattatagtagacTGTcgctggtatgtggccaatg	cattggccacataccagcgACAgtctactataatgtattgcca
I56D	tggcaatacattatagtagacGATcgctggtatgtggccaatg	cattggccacataccagcgATCgtctactataatgtattgcca
I56E	tggcaatacattatagtagacGAGcgctggtatgtggccaatg	tggcaatacattatagtagacGATcgctggtatgtggccaatg
D187A	gacttcgtaaaagtaGCAgatctctccgcccctatttac	gtaaataggggcggagagatcTGCtacttttacgaagtc
D242A	gtggcgtactgtcgatGCAttctgggataactggccccacct	aggtggggccagttatcccagaaTGCatcgacagtacgccac

Table B. Non-characterised protein sequences included in the phylogenetic analysis.

Uncharacterised protein[a]	Characterised homologue	Sequence identity
H1Y2N6 *Mucilaginibacter paludis*	*Cp*Arap27	72 %
W6UJJ5 *Pedobacter* sp	*Cp*Arap27	67 %
E2NCR7 *B. cellulosilyticus*	*Cp*Arap27	63 %
J1KQR8 *Flavobacterium* sp	*Cp*Arap27	79 %
A5FFA1 *F. johnsoniae*	*Cp*Arap27	57 %
B5I744 *S. sviceus*	*Cj*Aga27A	49.8 %
L7ETW0 *S. turgidiscabies*	*Se*MelA	57.9 %
T1UZ55 *A. mediterranei*	*Cm*Aga27A	48.6 %
W4NUI7 *B. caribensis*	*Cj*Aga27A	38.5 %
K7D6T7 *P. troglodytes*	*Hs*a-GAL	98 %
F1PGD0 *C. familiaris*	*Hs*a-GAL	85 %
G7XCA9 *A. kawachii*	*Tr*Agl1	54 %
I8ADE3 *A. oryzae*	*Tr*Agl1	57 %
A1D0A3 *N. fischeri*	*An*AglA	66 %
B8N306 *A. flavus*	*An*AN7152.2	62.5 %
B8MWJ5 *A. flavus*	*An*AglA	69 %
G5D7B5 *A. niger*	*An*AglA	97 %
E9FBI1 *M. anisopliae*	*An*AglA	47 %
Q9HFB1 *Acremonium* sp	*An*AglA	45 %

[a]Proteins are identified by UNIPROT code and organism name.

[b]Other proteins that have not biochemically characterised, but were included in the tree because a crystal structure is available, are Q9KBQ5 (*B. halodurans*), Q5LFG6 (*B. fragilis*) and Q5L7T7 (*B. fragilis*); these proteins are indicated on the phylogenetic tree along with their PDB codes.

Supporting figures

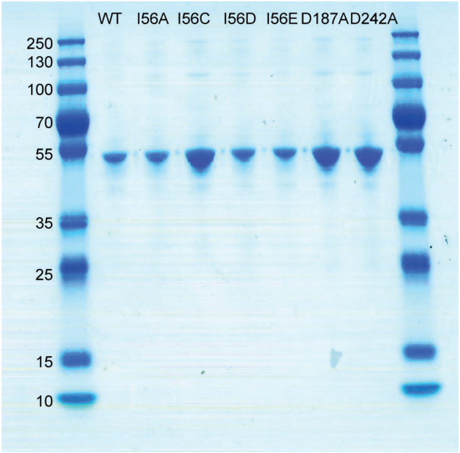

Fig A. Coomassie blue-stained gel obtained by SDS-PAGE of purified wild-type and mutant forms of *Cp*Arap27. Wild-type (WT) *Cp*Arap27 and all variants were purified on previously unused nickel resin to comparable purity and homogeneity. The size of the protein without signal peptide was predicted to be 48.0 kDa.

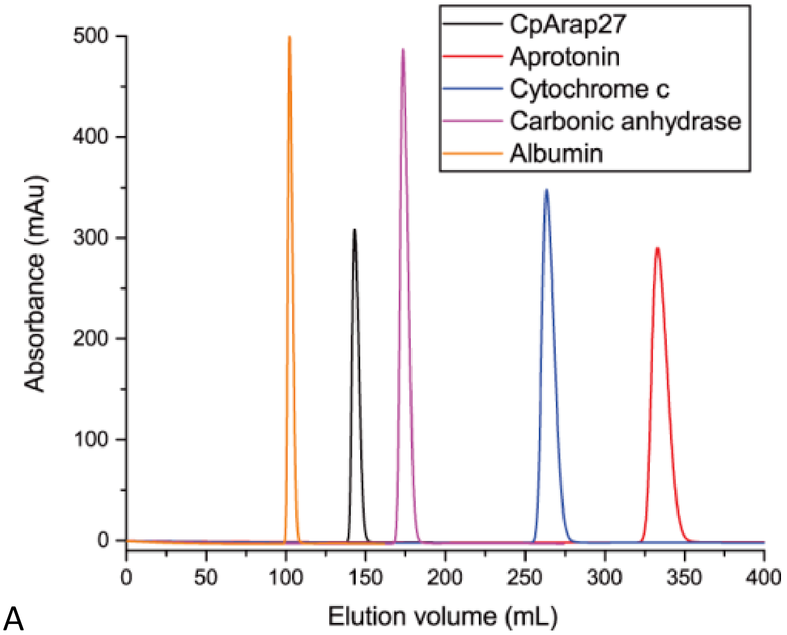

A

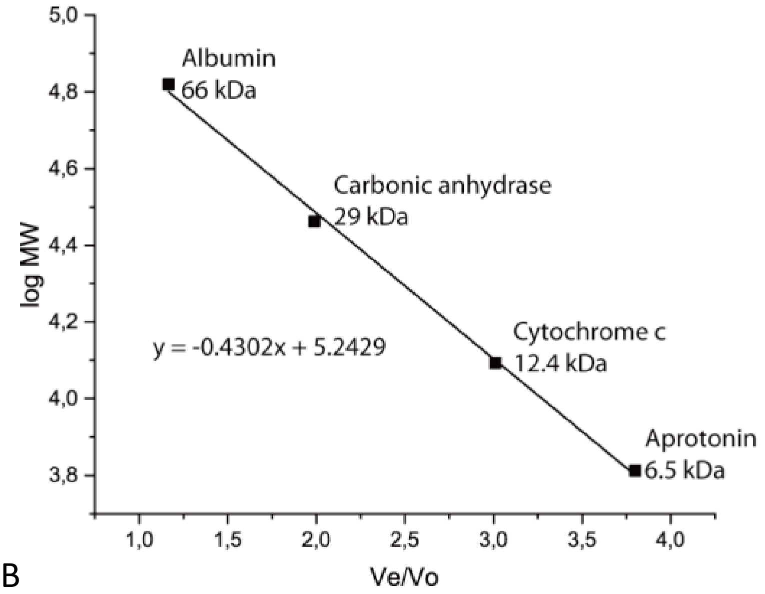

B

Fig B. Size-exclusion chromatography of *Cp*Arap27. A. Overlay of chromatograms of molecular mass standards and *Cp*Arap27. *Cp*Arap27 eluted as a single, sharp peak, with no evidence of oligomers at the void volume. B. Calibration curve. The observed peak elution volume of *Cp*Arap27 was 135 mL, corresponding to a v_e/v_o value of 1.324 and a molecular mass of 47 kDa (*cf.* Fig. S1).

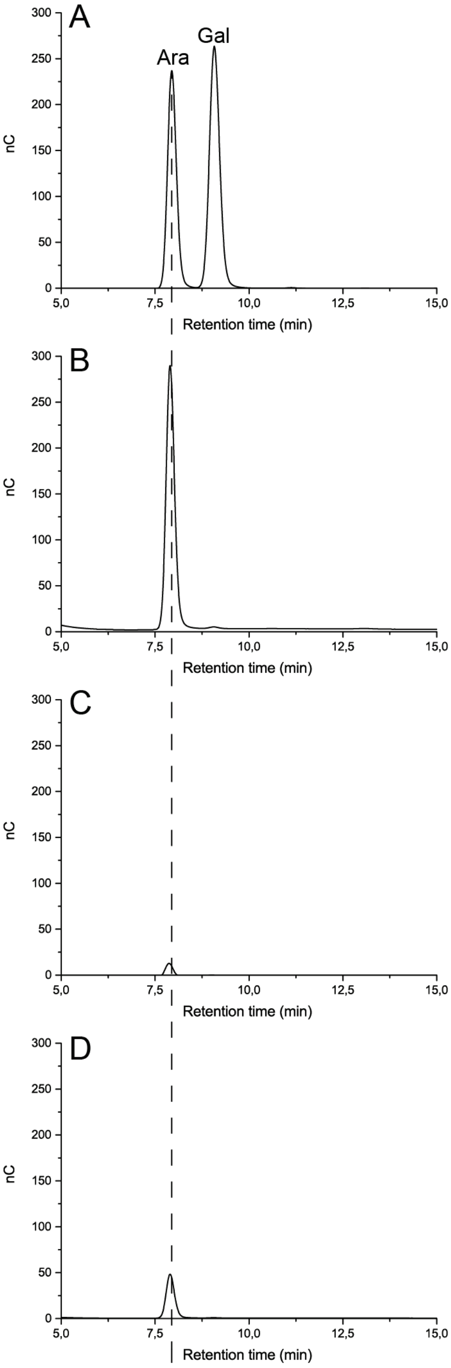

Fig C. HPAEC-PAD of products release from larch wood arabinogalactan by wild-type and mutant forms of *Cp*Arap27. HPAEC of arabinose and galactose standards (A), against which are compared the reaction products of three enzyme variants: wild-type (B), I56D (C) and I56E (D). Arabinose is the sole reaction product released from larch wood arabinogalactan in all cases.

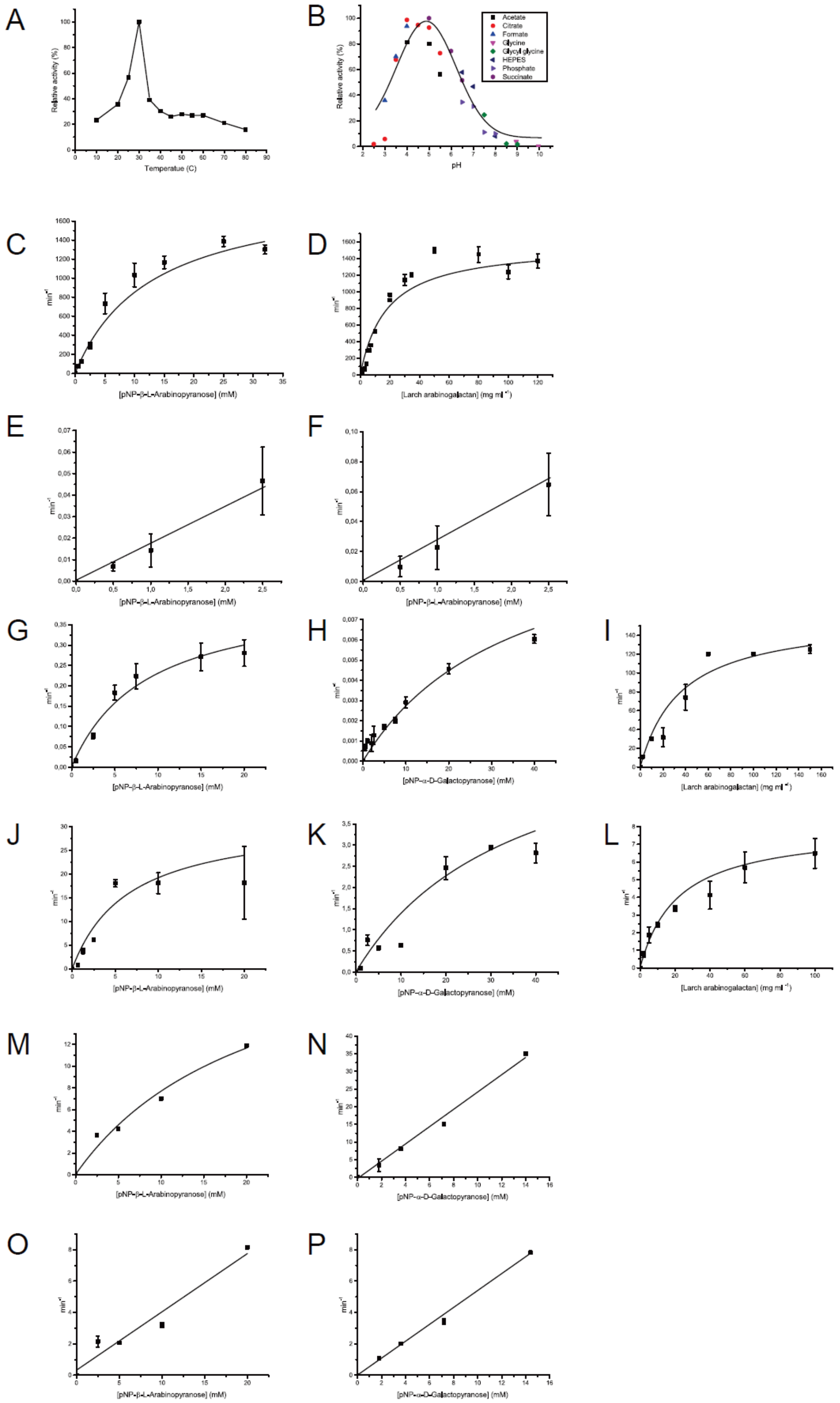

Fig D. Biochemical analysis of wild-type and mutant forms of *Cp*Arap27. (A) Optimum temperature conditions determined for the wild-type enzyme, using a stopped assay to analyse hydrolysis of the pNP-β-L-Ara*p* substrate. (B) pH optimum of the wild-type enzyme, determined using the same assay,

displayed with a Gaussian fit of the data. (C and D) Kinetic analysis of the wild-type enzyme against pNP-β-L-Ara*p* and larch wood arabinogalactan, respectively. (E and F) Kinetic analysis of the D187A and the D242A catalytic mutants, respectively, against pNP-β-L-Ara*p*. (G, H and I) Kinetic analysis of the I56D variant against pNP-β-L-Ara*p*, pNP-α-D-Gal*p* and larch wood arabinogalactan, respectively. (J, K and L) Kinetic analysis of the I56E variant against pNP-β-L-Ara*p*, pNP-α-D-Gal*p* and larch wood arabinogalactan respectively. (M and N) kinetic analysis of the I56C variant against pNP-β-L-Ara*p* and pNP-α-D-Gal*p* respectively. (O and P) kinetic analysis of the I56A variant against pNP-β-L-Ara*p* and pNP-α-D-Gal*p* respectively. For kinetic analysis of hydrolysis of the pNP-β-L-Ara*p* substrate, a continuous assay reading absorbance at 410 nm was used. For kinetic analysis of the cleavage of arabinose side chains from larch wood arabinogalactan, a linked galactose dehydrogenase assay was used, reading absorbance at 340 nm. Full descriptions of both assays are given in Experimental Procedures. All graphing and curve fitting was performed using Origin 9.1.
